# Editorial: Recent Developments in Therapies and Diagnostic Tools for Melanoma and Non-melanoma Skin Cancer

**DOI:** 10.3389/fmed.2020.613152

**Published:** 2020-11-12

**Authors:** Taku Fujimura, Yasuhiro Fujisawa, Atsushi Otsuka, Nikolas K. Haass

**Affiliations:** ^1^Department of Dermatology, Tohoku University Graduate School of Medicine, Sendai, Japan; ^2^Department of Dermatology, University of Tsukuba, Tsukuba, Japan; ^3^Department of Dermatology, Kyoto University Graduate School of Medicine, Kyoto, Japan; ^4^The University of Queensland Diamantina Institute, The University of Queensland, Brisbane, QLD, Australia

**Keywords:** biomarker (BM), immune checkpoint antibodies, dermoscopy (DS), skin imaging, T-cell lymphoma cutaneous, sentinel lymph node biopsy (SLNB), deep-learning (DL), convolutional neural network (CNN)

## Updates on Biomarkers for Immune Checkpoint Therapy of Advanced Melanoma

In the past decade, there has been a paradigm shift for the treatment of skin cancer, especially of advanced melanoma, due to the unprecedented success of MAPK pathway and immune checkpoint inhibitors (MAPKi, ICi). In this Research Topic, we focus on the latter. The most commonly used ICi are monoclonal antibodies targeting cytotoxic T-lymphocyte-associated protein 4 (CTLA-4; ipilimumab), programmed cell death 1 (PD-1; nivolumab and pembrolizumab) and, more recently, programmed death-ligand 1 (PD-L1; atezolizumab, durvalumab, and avelumab). In addition to multiple described resistance mechanisms to ICi (1), the lack of reliable biomarkers for both drug efficacy and immune-related adverse events (irAEs) has been a major clinical concern and is reviewed here extensively [Kambayashi et al.; Nakamura (a)]. Importantly, although many predictive biomarkers for both ICi tumor response and irAEs have been identified, no single biomarker has shown to be predictive on its own; therefore, a combination of multiple biomarkers should be utilized [Kambayashi et al.; Nakamura (a)]. Furthermore, irAEs and tumor response can be interconnected, but often are not; thus, irAEs or biomarkers for irAEs cannot be reliably used as biomarker for tumor response [Kambayashi et al.; Nakamura (a)]. Further studies are needed to improve biomarkers for predicting the efficacy of ICi treatment [Kambayashi et al.; Nakamura (a)]. To this end, Kümpers et al., show that PD-L1 expression on primary tumors and melanoma metastases is not associated with the clinical response of anti-PD1 antibodies, while several previous studies suggest an association of PD-L1 status and response to anti-PD1 antibodies (2). Instead, immune cell infiltration in the primary melanoma, measured by the Immunoscore, was associated with a significantly improved response to ICi in terms of increased overall survival (Kümpers et al.). Another research paper in this Research Topic suggests that baseline serum levels of CXCL5, which have been reported previously as a biomarker for autoimmune disease, could be a predictive marker for the efficacy of anti-PD1 antibodies (Fujimura, Sato et al.). While ICi were initially only used for definitive therapy, the field has quickly moved to adjuvant and, more recently, to neoadjuvant therapy (3). Combination of nivolumab plus ipilimumab (N+I) is amongst the most effective therapies against both BRAF-mutated and BRAF-wildtype advanced melanoma, but leads to a high frequency of irAEs [Nakamura (a)]. Fujimura, Kambayashi et al. show in a case report that N+I combination therapy for BRAF-mutated advanced melanoma before primary tumor resection strikingly increased CD8+ cytotoxic T cells in the primary tumor, leading to induced anti-melanoma immune response in metastases in six different organs, but also induced serious AEs after administration of N+I combination therapy (4). In addition, previous reports suggest that the efficacy of ipilimumab among patients with anti-PD1 antibody-resistant melanoma is extremely low after objective tumor progression (5). In summary, the optimization of immunotherapy using ICi is still challenging, but rapidly developing, and it is exciting to see how the landscape of melanoma biomarkers has changed within a decade (6).

## Updates on Systemic Therapies for Non-Melanoma Skin Cancers

On the heels of the success of systemic therapies for melanoma, similar approaches are being tested in non-melanoma skin cancers (NMSC). Tanese et al. review systemic therapy options, including hedgehog inhibitors for basal cell carcinoma (BCC), EGFR inhibitors and ICi for cutaneous squamous cell carcinoma (cSCC), HER2 antagonists for extramammary Paget's disease (EMPD), ICi for Merkel cell carcinoma (MCC), and experimental approaches for skin adnexal carcinomas. They conclude that, emerging molecular targeting therapies are not necessarily effective for all NMSC patients. Development of further treatment options for NMSC is required, especially for rare forms of NMSC, such as skin adnexal carcinomas (Tanese et al.). A retrospective case series by Hiura et al. of 13 patients with unresectable cSCC demonstrates the potential advantage of continued chemotherapy after concurrent chemoradiotherapy (CCRT), which will be validated in a future study. Hidaka et al. discuss in a thought-provoking review the role that Aryl Hydrocarbon Receptor (AHR) plays in carcinogenesis and maintenance of skin cancers. AHR is a key modulator of UVR- and carcinogenic chemical-induced skin carcinogenesis and is also associated with the efficacy of MAPKi and ICi in melanoma (Hidaka et al.). Thus, the authors propose that the AHR system may provide a putative target for prevention and therapy of skin cancer (Hidaka et al.). Oka and Miyagaki review novel and future therapies for advanced Mycosis fungoides and Sézary syndrome covering a wide range of different drug classes. Most approaches showed limited efficacy. Thus, the authors recommend personalized therapy and call for creatively designed international clinical trials (Oka and Miyagaki).

## Updates on Diagnostic Tools for Melanoma and Non-Melanoma Skin Cancer

Dermoscopy has become an indispensable diagnostic tool for pigmented and unpigmented cutaneous lesions (7). Kato et al. review the role of dermoscopy in the diagnosis of melanoma and non-melanoma skin cancers including BCC, sebaceous carcinoma, actinic keratosis, Bowen's disease, cSCC, MCC, EMPD, and angiosarcoma. Oh et al. take skin imaging further by discussing the utility of ultrasound imaging, optical coherence tomography, confocal microscopy, and two-photon microscopy as diagnostic tools. Especially a combination of tools is advised to allow for highest resolution and highest imaging depth, which are usually reciprocal (Oh et al.). The principle of these devices is to analyze signals reflected or scattered from the skin. Indeed, the fact that autofluorescent structures within the skin (e.g., elastic fibers) can be co-imaged with highly crystalline triple-helix structures (e.g., collagen) utilizing the second harmonic generation phenomenon, which then can be quantified (8). Oh et al. suggest that the development of fluorescent probes will further improve the utility of these tools for the diagnosis and treatment of skin lesions. Fujisawa et al. take this further and discuss the strengths and limitations of a deep-learning technology using a convolutional neural network (CNN) for skin tumor diagnosis. They conclude that AI classifiers have dramatically improved over the last years and still keep improving and thus may gain sufficient sensitivity and specificity to bear the screening burden for detecting malignant skin tumors (Fujisawa et al.). Importantly, they emphasize that the advent of AI-based skin cancer diagnostic should be considered a useful assistance, rather than a threat to dermatologists (Fujisawa et al.).

Nakamura (b) discusses the new role of sentinel lymph node biopsy (SLNB) for invasive melanoma post DeCOG-SLT and MSLT–II, in the context of modern adjuvant and neoadjuvant therapy approaches.

Fujii and Kanekura review the methods for diagnosis of early stage T-Cell Lymphoma. They demonstrate that next-generation sequencing not only detects TCR clonality with superior sensitivity over conventional methods and is therefore better to diagnose early Mycosis fungoides, but also allows for temporal tracking of specific TCR clones and therefore better for assessment of progression or recurrence (Fujii and Kanekura).

## Author Contributions

TF and NH wrote the manuscript. All authors provided intellectual input to the editorial.

## Conflict of Interest

The authors declare that the research was conducted in the absence of any commercial or financial relationships that could be construed as a potential conflict of interest.
